# Posture alteration as a measure to accommodate uneven ground in able-bodied gait

**DOI:** 10.1371/journal.pone.0190135

**Published:** 2017-12-27

**Authors:** Soran Aminiaghdam, Reinhard Blickhan, Roy Muller, Christian Rode

**Affiliations:** Department of Motion Science, Institute of Sport Sciences, Friedrich Schiller University Jena, Jena, Thuringia, Germany; Fondazione Santa Lucia Istituto di Ricovero e Cura a Carattere Scientifico, ITALY

## Abstract

Though the effects of imposed trunk posture on human walking have been studied, less is known about such locomotion while accommodating changes in ground level. For twelve able participants, we analyzed kinematic parameters mainly at touchdown and toe-off in walking across a 10-cm visible drop in ground level (level step, pre-perturbation step, step-down, step-up) with three postures (regular erect, ~30° and ~50° of trunk flexion from the vertical). Two-way repeated measures ANOVAs revealed step-specific effects of posture on the kinematic behavior of gait mostly at toe-off of the pre-perturbation step and the step-down as well as at touchdown of the step-up. In preparation to step-down, with increasing trunk flexion the discrepancy in hip−center of pressure distance, i.e. effective leg length, (shorter at toe-off versus touchdown), compared with level steps increased largely due to a greater knee flexion at toe-off. Participants rotated their trunk backwards during step-down (2- to 3-fold backwards rotation compared with level steps regardless of trunk posture) likely to control the angular momentum of their whole body. The more pronounced trunk backwards rotation in trunk-flexed walking contributed to the observed elevated center of mass (CoM) trajectories during the step-down which may have facilitated drop negotiation. Able-bodied individuals were found to recover almost all assessed kinematic parameters comprising the vertical position of the CoM, effective leg length and angle as well as hip, knee and ankle joint angles at the end of the step-up, suggesting an adaptive capacity and hence a robustness of human walking with respect to imposed trunk orientations. Our findings may provide clinicians with insight into a kinematic interaction between posture and locomotion in uneven ground. Moreover, a backward rotation of the trunk for negotiating step-down may be incorporated into exercise-based interventions to enhance gait stability in individuals who exhibit trunk-flexed postures during walking.

## Introduction

In addition to investigating a locomotor system operating in steady-state conditions, the study of its behavior when coping with perturbations can lead to further identification of the system properties [1]. During locomotion, human must not only ensure a forward progression in accordance with dynamic equilibrium, but is also required to continuously cope with perturbations—such as postural changes, terrain variations, obstacles, drops, etc.—in an anticipatory fashion through coordinated interactions between different body segments [2, 3]. Maintaining dynamic stability across uneven ground can be a critical issue to locomotion. The gait is assumed stable if it returns to a periodic trajectory after being exposed to a perturbation and it can be considered robustly stable if it can recover from large perturbations [4]. Experimentally imposed trunk flexion [5–7] and changing ground level [8, 9] have been proposed as two types of perturbations to human locomotion.

The trunk plays a key role in human locomotion. It may perform as a reference in the control of posture and movement in upright gait [10, 11]. Stabilizing the trunk, an unstable inverted-pendulum positioned over the hips [12, 13], is a crucial locomotor task. Due to its large mass, the trunk orientation has considerable effects on the ground reaction force (GRF) [14] and the center of mass (CoM) trajectory [5, 7]. The relative position of the hip with respect to the CoM determines the effective leg (connecting the hip and the center of pressure [CoP]) function [5, 15]. A forward inclination of the trunk can be utilized to generate a greater forward propulsion through the hip in various forms of locomotion involving fast walking, uphill gait [16] and stepping up [17]. Furthermore, a backward rotation of the trunk has been observed during step-down, possibly to regulate the whole-body angular momentum [9]. In [5], we speculated that a dynamic backward trunk rotation during trunk-flexed walking may reduce the vertical CoM oscillation in walking across uneven ground. If this speculation can be confirmed, it may find clinical applications benefitting individuals exhibiting trunk-flexed posture and impaired postural control [18, 19].

Bending the trunk forward in level walking leads to an anterior shift of the CoM with respect to the hip. This causes a shorter effective leg at toe-off (TO) than at touchdown (TD; heel strike) [5, 20], and this intra-limb asymmetry increases with trunk flexion [5]. Despite an unchanged effective leg length [5], trunk-flexed gait is associated with a posterior shift of the pelvis relative to the CoP [5, 20], together with crouched legs during the stance phase [5, 7].

While many aspects of human locomotion involving the mechanisms of postural control in the context of unexpected changes in surface conditions [21–26], the effect of trunk posture on gait [5–7, 18, 27–31], and the kinematic and kinetic adjustments during crossing uneven ground [8, 9, 32–36] have been extensively studied, little is known about kinetic and kinematic adaptions in human locomotion over uneven ground with altered trunk orientation. In a recent study [37] focusing on kinetic adjustments in walking across uneven ground, we found reduced between-step variations in the GRF patterns with increasing trunk flexion. We expect the compensatory kinematic strategies that enable the observed reduced between-step kinetic effects when walking with trunk-flexed gaits across uneven ground. Coping with such gait conditions is likely to present different challenges compared to upright postures. Understanding these challenges is of clinical interest as age or some pathological conditions can alter the trunk posture and the adaptive capacity of human locomotor system [38–42].

Considering an altered dynamics of the trunk-flexed gaits from regular upright walking [5–7, 18, 27, 28, 31], the context-specific kinetic and kinematic adaptations during walking and the intra-limb kinetic and kinematic asymmetries in leg function at TD and TO as a result of an increased sagittal trunk flexion [5], this study aims at examining the adaptive locomotor kinematic behavior in perturbed steps (10 cm visible drop; level step, pre-perturbation step, step-down, step-up) while walking with three postures (regular erect, with ~30° and ~50° trunk flexion from the vertical). We expect step-specific effects of imposed trunk posture on kinematic parameters of human walking, with more pronounced adaptations at TO since a posterior shift of the hip relative to the CoM during trunk-flexed gaits leads to a shorter effective leg at TO than at TD and correspondingly to a flatter leg angle at TD and a steeper one at TO. Furthermore, we hypothesize that the kinematic adaptations across steps would be posture-dependent, i.e. more pronounced kinematic adjustments during trunk-flexed gaits that may be necessary for maintaining balance, and that these adaptations would affect the vertical oscillation of the CoM. Specifically, we hypothesize that participants exploit a backward rotation of the trunk during step-down to reduce effects of the step-down on the CoM height. Finally, we expect a robustly stable walking, i.e. an immediate restoration of the kinematic parameters at the end of the step-up following the step-down, despite alteration in the trunk posture owing to the adaptive capacity of the locomotor system in young healthy participants.

## Materials and methods

### Participants

Twelve (six males, six females) healthy volunteers (mean ± SD; age = 26 ± 3.35 years, height = 169.75 ± 7.41 cm, mass = 65.08 ± 8.07 kg) with no history of orthopedic (leg length discrepancy, joint fracture, joint laxity, arthritis), musculoskeletal and neurologic disorders participated in this study. Lower limb range of motion was not assessed. A consent form was signed by each participant before participation. The experimental protocol was approved by the local Ethics Committee of the Friedrich Schiller University Jena (3532-08/12) and carried out in accordance with the Declaration of Helsinki.

### Experimental design and measurements

Kinematic data was collected using eight infra-red Qualisys motion capture cameras (MCU1000, Qualisys, Gothenburg, Sweden) sampling at 240 Hz. GRFs during walking were measured at 1000 Hz using three consecutive force platforms (9285BA, 9281B, 9287BA, Kistler, Winterthur, Switzerland), embedded in the middle portion of a 12 m-long walkway. Kinematics and GRF data were synchronized by using the Kistler’s external trigger and BioWare data acquisition software (Kistler Instrument AG, Winterthur, Switzerland). Data collection was conducted at the Biomechanical Laboratory of the Sports Institute within Friedrich Schiller University Jena. Spherical retro-reflective surface markers (14 mm) were used to track the motion of the body. A thirteen-body segment model [5] was defined using 21 markers. The markers were placed on the following bony landmarks: fifth metatarsal heads, lateral malleoli, lateral epicondyles of femurs, greater trochanters, anterior superior iliac spines, posterior superior iliac spines, L5-S1 junction, lateral humeral epicondyles, wrists, acromioclavicular joints, seventh cervical spinous process and middle of the forehead.

**Fig 1:**
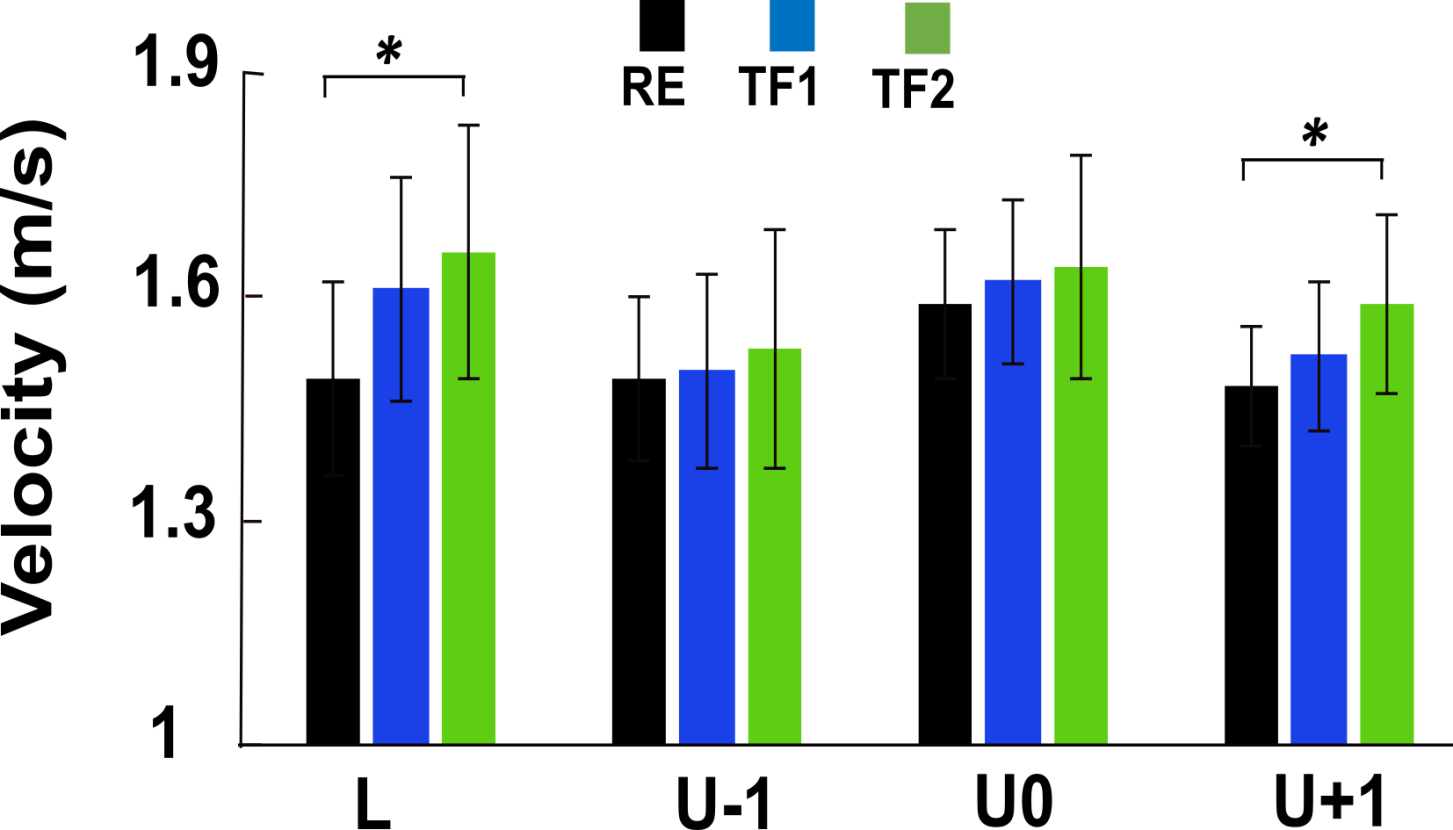
Gait velocity across steps and postures. Simple main effect analysis showed that participants walked with an increased velocity during gait with 50° of trunk flexion (TF2) in unperturbed step (p = 0.02) and step-up (p = 0.03) as compared to the gait with regular upright posture (RE); however, there were no between step differences when walking with RE (p = 0.51), TF1 (p = 0.55) and TF2 (p = 0.11). Error bars denote standard deviation. RE, regular erect trunk; TF1, ~30° trunk flexion; TF2, ~50° trunk flexion; L, unperturbed level step; U-1, pre-perturbation step; U0, step-down; U+1, step-up.

**Fig 2:**
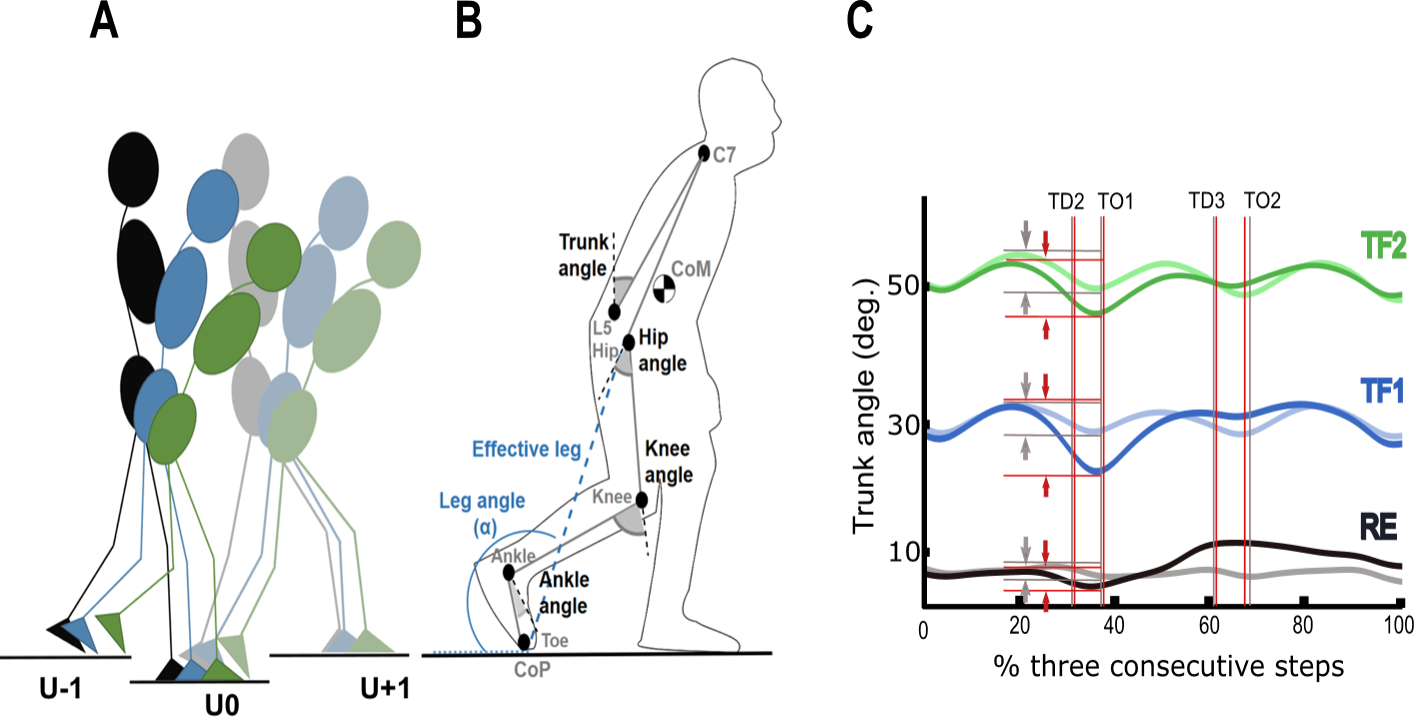
Human locomotion diagram and trunk angle trajectories. (A) Side view of the instrumented walkway with three consecutive force plates. The second force plate (step-down) was lowered 10 cm during uneven walking with RE, TF1 and TF2 conditions. (B) Illustration of the definitions of the trunk angle as well as hip, knee, and ankle joint angles, the effective leg and the leg angle as used in this study. (C) The trunk kinematics in the sagittal plane across three level steps (blurred curves) and three uneven steps (solid curves) with regular erect (RE, black), ~30° of trunk flexion (TF1, blue) and ~50° of trunk flexion (TF2, green) during walking. The vertical grey and red lines represent TD and TO instants pertaining to the three consecutive steps during level and uneven walking, respectively. The horizontal grey and red lines highlight the maximum of the trunk angle in the step ‘U-1’ and the minimum of the trunk angle in the step ‘U0’ for each walking postures, respectively. L, unperturbed level step; U-1, pre-perturbation step; U0, step-down; U+1, step-up; CoM, center of mass; α, leg angle; CoP, center of pressure; TD, touchdown; TO, toe-off.

Participants were instructed to walk at their self-selected normal walking speed (Fig 1) (with no restriction on the arm movements) across two experimental ground conditions involving a level walkway and a walkway with a 10 cm drop for each of the three conditions: self-selected regular erect trunk alignment (RE), 30° (TF1) and 50° (TF2) (Fig 2). One height-variable force plate at the site of the second contact and two ground-level force plates at the site of the first and third contacts were set (Fig 2A). After walking on the unperturbed level track, the variable-height force plate was lowered by 10 cm and participants walked along the uneven walkway. To determine the most consistent trunk posture across participants, trunk flexion was achieved by bending from the hips [5, 7, 37]. Trunk angle was defined by the angle sustained by the line connecting the L5 marker (midpoint between the L5–S1 junction) and the C7 marker (seventh cervical spinous process) with respect to the vertical axis of the lab coordinate system (Fig 2B) [5, 9, 37]. A co-examiner compared trunk angles (TF1 and TF2) visually with adjustable-height cardboard templates prior to performing of each trial and during gait along the walkway. The templates, drawn with lines displaying target trunk flexion angles TF1 and TF2, were hung on a wall parallel to the walkway: one at the beginning and the other one in the middle of walkway [5, 7, 37]. Participants were encouraged to walk along the walkway to accommodate to the locomotion conditions and secure step onto the force plates. The dominant lower limb was defined based on participants’ verbal report of which limb they use to kick a soccer ball [43]. To simulate the natural situation of arbitrary step-down with respect to limb dominance [44], we defined a left-right-left sequence thus making sure that some participants stepped down with the dominant limb (n = 7), some not (n = 5) [9]. Due to organizational reasons, level and uneven setups as well as repetitions of trunk orientations were not randomized, but the sequence of flexed trunk orientations were randomized per participant. While maintaining each gait posture, the participants performed eight successful trials in which each single force plate was cleanly struck by one foot.

### Parameters of interest

The ensemble average of following parameters of interest, in addition to their angular trajectories throughout stance phase of each individual step, were determined in the sagittal plane: 1) hip, knee and ankle joint angles (Fig 2B) at the instants of TD and TO; 2) effective leg length, defined as the length between the hip and CoP (Fig 2B), at the instants of TD (EL_*TD*_) and TO (EL_*TO*_); 3) vertical position of the CoM at the instants of TD (CoM_*TD*_) and TO (CoM_*TO*_) relative to the ground determined by the body segmental analysis method relative to the laboratory coordinate system [45, 46]; 4) leg angle, angle between effective leg and ground (Fig 2B), at the instants of TD (α_*TD*_, angle of attack) and TO (α_*TO*_) was calculated with respect to the negative x-axis. A vertical GRF threshold of 0.03 body weight was used to determine the instants of TD and TO at each contact [5]. The effective leg length and CoM were both normalized to the distance between the greater trochanter marker and the lateral malleoli marker at the instant of TD. Backward rotation during step-down was calculated as the difference of the maximum of the trunk angle in ‘U-1’ and the minimal trunk angle in ‘U-0’ (Fig 2C).

### Data processing and statistics

Kinetic and kinematic data of all successful trials were analyzed using custom written Matlab (Mathworks Inc., MA, USA) code. The raw coordinate data were filtered using a fourth-order low-pass, zero-lag Butterworth filter with 12 Hz cutoff frequency [5, 37].

Prior to analysis Levene's test and Shapiro-Wilk test were performed to examine equality of variance and normality of distribution, respectively. We analyzed all data sets using a two-way repeated measures ANOVA to examine the effects of the posture (RE, TF1 and TF2) and step (unperturbed step ‘L’ in level ground; pre-perturbation step ‘U-1’, step-down ‘U0’ and step-up ‘U+1’ across uneven ground) on the vertical position of the CoM, the effective leg length and angle, and the lower limb joints (hip, knee and ankle) at TD and TO instants. In case of a significant interaction, simple main effects were used to compare walking postures across each step, as well as across steps while walking with each individual posture using one-way ANOVA and post-hoc comparisons with Bonferroni adjustments for multiple comparisons. In case of a non-significant interaction, the main effects of the posture and step were evaluated on each dependent variable of interest. Where Mauchly’s test indicated a violation of sphericity, *p*-values and degrees of freedom were corrected using the Greenhouse–Geisser correction factor. Furthermore, paired t-tests (using mean values per subject) were used to compare backward rotation of the trunk in level and perturbed (step-down) walking for each trunk inclination. All statistical analyses were performed with SPSS Statistics 21 (IBM Corporation, New York, NY, USA). The statistical significance level of all tests was set to *p* = 0.05.

## Results

The data analyzed includes 576 trials with a total of 1728 step cycles. Participants were successful in maintaining their stability (no falls) on every trial while crossing the level and uneven ground. Table 1 shows the mean trunk angles at TD and TO across steps while maintaining trunk postures. Mean trunk backward rotations during step down were significantly higher than those for level steps across all gait conditions (Fig 2C). The backward rotation in RE gait increased from 3.5 ± 0.8° during level walking to 5.7 ± 1.9° during step-down (t = 3.89, p = 0.003), in TF1 from 4.8 ± 3.4° to 14.9 ± 10.9° (t = 2.95, p = 0.01) and in TF2 gait from 5.6 ± 2.4° to 9.9 ± 3.4° (t = 4.62, p = 0.001).

**Table 1:** Means and standard deviations of kinematic parameters.

	Step	Posture			p-value/F-value		
					ES		
		RE	TF1	TF2	Posture	Step	Posture × Step
**Trunk**_***TD***_ **(deg)**	L	6.2±3.4	30.4±6.2	47.3±6.9			
	U-1	6.1±3.3	29.8±6.3	46.6±5.2			
	U0	5.2±5.6	26.2±6.5	43.8±5.9			
	U+1	12.6±4.4	32.6±6.7	48.9±3.9			
**Trunk**_***TO***_ **(deg)**	L	5.0±3.4	29.7±5.1	45.9±7.6			
	U-1	5.2±5.4	23.9±10.1	43.1±3.9			
	U0	11.5±5.0	24.2±13.1	49.1±6.2			
	U+1	7.5±5.4	27.0±11.3	46.2±4.0			
**Normalized CoM**_***TD***_	L	1.12±0.02	1.10±0.02	1.08±0.02	0.00/120	0.00/144	0.00/5.96
	U-1	1.12±0.02	1.07±0.08	1.07±0.02			
	U0	1.16±0.03	**1.14±0.03**	**1.11±0.03**^a^	0.95	0.96	0.49
	U+1	**1.06±0.02**	1.05±0.02	**1.04±0.03**			
**Normalized CoM**_***TO***_	L	1.12±0.02	1.10±0.02	1.08±0.02	0.00/42.5	0.00/105	0.69/0.64
	U-1	1.05±0.03	1.02±0.03	1.01±0.03			
	U0	1.17±0.02	1.15±0.03	1.13±0.03	0.87	0.94	0.09
	U+1	1.11±0.04	1.11±0.06	1.08±0.06			
**Normalized EL**_***TD***_	L	1.14±0.03	1.16±0.03	1.16±0.03	0.01/6.24	0.00/15.1	0.04/3.97
	U-1	1.14±0.04	1.15±0.04	1.15±0.03			
	U0	1.14±0.02	1.14±0.03	1.13±0.03	0.51	0.71	0.39
	U+1	**1.08±0.02**	**1.10±0.03**	**1.10±0.02**			
**Normalized EL**_***TO***_	L	1.12±0.03	1.10±0.02	1.09±0.03	0.00/7.16	0.00/37.3	0.00/5.69
	U-1	1.07±0.02	1.04±0.01^a^	1.01±0.02^a,b^			
	U0	**1.15±0.03**	**1.15±0.03**	**1.14±0.03**	0.54	0.86	0.48
	U+1	1.10±0.03	**1.09±0.04**	**1.07±0.05**			
**Hip**_***TD***_ **(deg)**	L	19.9±3.4	43.3±4.0^a^	53.5±8.2^a,b^	0.00/458	0.00/19.5	0.00/28.6
	U-1	20.4±3.9	37.4±8.7^a^	53.5±8.2^a,b^			
	U0	15.9±3.4	39.1±8.3^a^	51.0±6.9^a,b^	0.99	0.83	0.87
	U+1	**37.7±4.9**	42.7±4.4	53.4±7.2^a^			
**Hip**_***TO***_ **(deg)**	L	-12.4±5.0	11.9±7.4^a^	24.6±9.9^a,b^	0.00/145	0.33/1.24	0.00/7.89
	U-1	-13.0±8.0	11.0±9.2^a^	24.9±9.6^a,b^			
	U0	-8.0±4.1	11.3±8.9^a^	20.6±8.5^a^	0.97	0.23	0.66
	U+1	-13.1±6.7	11.7±8.4^a^	22.9±9.2^a^			
**Knee**_***TD***_ **(deg)**	L	9.6±4.0	10.8±3.5	10.7±5.3	0.00/9.37	0.00/31.7	0.00/12.9
	U-1	9.7±5.3	10.7±5.6	12.2±5.7			
	U0	14.1±5.6	15.6±6.0	16.3±5.8	0.70	0.88	0.76
	U+1	**29.2±5.4**	**22.8±8.4**	**21.1±8.1**			
**Knee**_***TO***_ **(deg)**	L	40.5±6.3	45.7±6.5	50.4±6.1^a^	0.00/9.67	0.00/31.1	0.00/5.27
	U-1	51.5±10.6	61.4±9.2	70.6±7.0^a^			
	U0	**30.7±3.6**	**30.2±4.5**	**32.9±4.0**	0.70	0.88	0.56
	U+1	**37.2±10.4**	**43.6±12.6**	**50.2±7.0**^a^			
**Ankle**_***TD***_ **(deg)**	L	-1.6±2.3	2.1±2.2^a^	2.7±1.9^a^	0.15/2.41	0.88/0.21	0.00/3.72
	U-1	-1.5±3.0	1.4±2.5	2.6±2.5^a^			
	U0	-2.0±10.9	-4.7±15.2	**-8.5±17.3**	0.37	0.05	0.48
	U+1	1.4±3.1	3.3±2.9	3.1±3.3			
**Ankle**_***TO***_ **(deg)**	L	-14.9±6.2	-11.5±2.5	-9.8±4.3	0.29/1.42	0.00/12.3	0.13/1.85
	U-1	-2.5±7.2	-2.1±4.4	-2.3±4.6			
	U0	-18.5±3.0	-16.6±3.2	-13.3±5.5	0.26	0.75	0.31
	U+1	-13.7±7.6	-11.8±3.0	-9.5±4.2			
**α**_***TD***_ **(deg)**	L	66.5±4.6	62.6±5.1	62.2±6.3	0.01/6.52	0.55/0.71	0.37/1.12
	U-1	65.0±4.7	63.1±4.4	59.9±7.9			
	U0	65.6±2.5	63.9±4.4	63.9±3.2	0.52	0.10	0.15
	U+1	64.6±2.9	64.0±2.8	63.4±2.7			
**α**_***TO***_ **(deg)**	L	117±2.7	116±5.9	116±7.3	0.09/2.92	0.00/33.0	0.84/0.44
	U-1	119±2.1	120±2.4	120±5.1			
	U0	120±2.8	119±4.6	120±3.0	0.32	0.84	0.06
	U+1	116±2.9	111±3.1	110±2.9			

The last three columns show the p-values/F-values and effect size (ES, partial eta squared) of the main effects of posture and step and, the posture×step interaction, respectively. In case of interaction effect, significant differences from RE and TF1 across each step are indicated with ‘a’ and ‘b’, respectively (p<0.05). Accordingly, shaded, bold and underlined values indicate the significant difference from the unperturbed step ‘L’, from the pre-perturbation step ‘U-1’ and from the step-down ‘U0’ (p<0.05), respectively, for each walking posture (N = 12). CoM, center of mass; TD, touchdown; TO, toe-off; EL_TD_, normalized effective leg length at TD; EL_TO_, normalized effective leg length at TO; α_TD_, leg angle at TD; α_TO_, leg angle at TO; RE, regular erect trunk; TF1, ~30° trunk flexion; TF2, ~50° trunk flexion; U+1, step-up.

**Fig 3:**
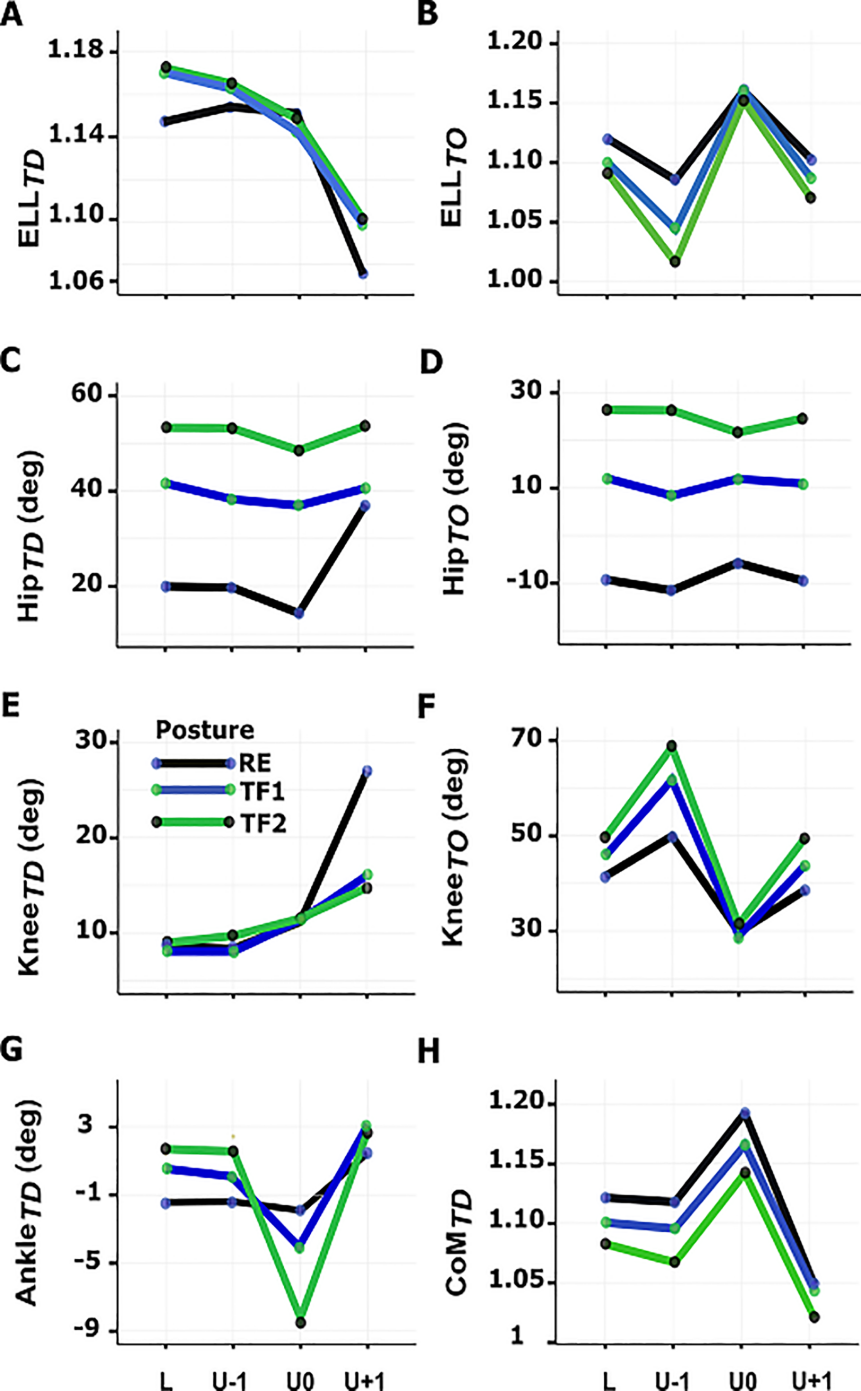
Posture×step interaction. (A)Normalized effective leg length at TD, (B) normalized effective leg length at TO, (C) hip position at TD, (D) hip position at TO, (E) knee position at TD, (F) knee position at TO, (G) ankle position at TD and (H) normalized CoM position at TD. (N = 12). RE, regular erect trunk; TF1, *~*30° trunk flexion; TF2, *~*50° trunk flexion; L, unperturbed level step; U-1, pre-perturbation step; U0, step-down; U+1, step-up.

Table 1 summarizes posture×step interactions and the main effects of posture and step on kinematic parameters. Two-way repeated measures ANOVAs indicated step-specific effects of the trunk orientation on normalized vertical position of the CoM at TD (CoM_*TD*_) (Fig 3H), normalized effective leg length at TD (EL_*TD*_) (Fig 3A) and TO (EL_*TO*_) (Fig 3B), hip angle at TD (Hip_*TD*_) (Fig 3C) and TO (Hip_*TO*_) (Fig 3D), knee angle at TD (Knee_*TD*_) (Fig 3E) and TO (Knee_*TO*_) (Fig 3F) and ankle joint at TD (Ankle_*TD*_) (Fig 3G).

**Fig 4:**
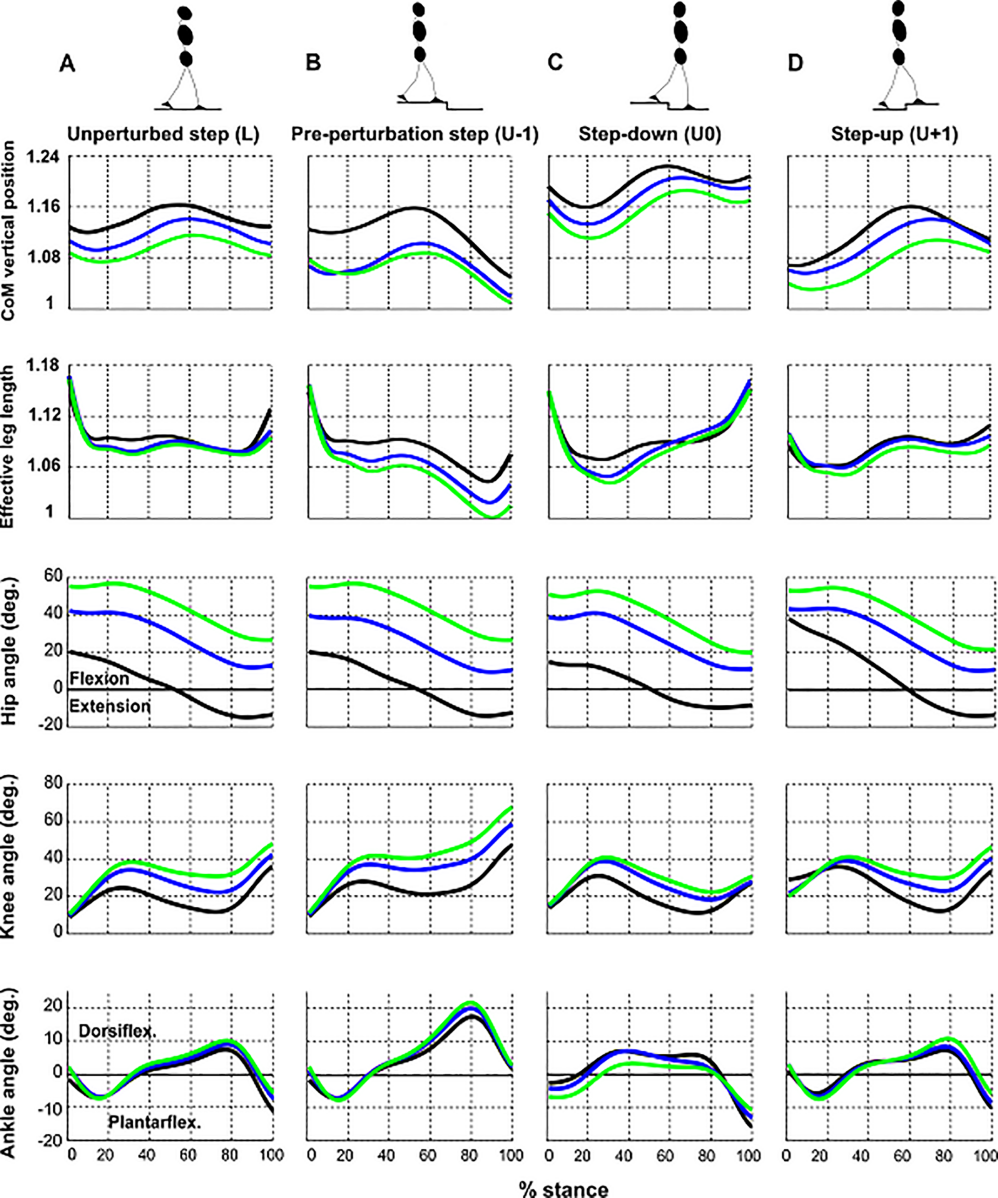
Normalized CoM, normalized effective leg length and lower limb joint angle trajectories. Shown are ensemble-averaged normalized vertical position of center of mass (CoM), normalized effective leg length, hip, knee, and ankle angles pertaining to (A) unperturbed step (L), (B) pre-perturbation step (U-1), (C) step-down (U0) and (D) step-up (U+1) in the sagittal plane during the stance phase for RE (black), TF1 (blue) and TF2 (green) (N = 12). RE, regular erect trunk; TF1, ~30° trunk flexion; TF2, ~50° trunk flexion.

Post-hoc tests revealed no significant differences of EL_*TD*_ and EL_*TO*_ between gait postures during unperturbed level step ‘L’ (Figs 3A, 3B and 4A, Table 1). In the pre-perturbation step ‘U-1’, while EL_*TD*_ exhibited no significant changes across gait postures and compared to the corresponding level steps, significantly lower EL_*TO*_ compared to the level steps was found in all gait postures with a decreased EL_*TO*_ by ~3% and ~6% from RE gait to 1.04 ± 0.02 and 1.01 ± 0.02 in TF1 and TF2 gaits, respectively (Figs 3A, 3B and 4B, Table 1). During the step-down ‘U0’, EL_*TD*_ remained relatively unchanged as compared to the corresponding level steps and showed no between gait posture differences. Trunk-flexed gaits (TF1 and TF2) demonstrated a significantly elongated effective leg at TO (EL_*TO*_) after step-down compared with corresponding values of both ‘L’ and ‘U-1’ steps with no between gait posture differences (Figs 3A, 3B and 4C, Table 1). Significantly shortened EL_*TD*_ in the step-up ‘U+1’compared to all preceding steps in all gait postures with no between gait posture differences was found (Figs 3A and 4D, Table 1). EL_*TO*_ demonstrated a significant increase in trunk-flexed gaits relative to the step ‘U-1’ and a significant decrease compared to the step ‘U0’ regardless of the trunk orientation (Figs 3B and 4D, Table 1).

The tests of simple main effects revealed that trunk-flexed gaits demonstrated an increased Hip_*TD*_ and Hip_*TO*_ across all steps with no between step differences except for the RE gait in the step ‘U+1’ where the hip flexion increased by ~18° compared to the step ‘L’ (Figs 3C, 3D and 4, Table 1).

For the Knee_*TD*_, no between step and between posture differences were found except for the step ‘U+1’ where the knee flexion dramatically increased in all gait postures compared to the preceding corresponding steps with no between posture differences (Figs 3E and 4, Table 1). In the step ‘L’, TF2 gait led to a significant increase of ~10° in Knee_*TO*_ compared to the RE gait (Figs 3F and 4A, Table 1). Significantly increased Knee_*TO*_ in the step ‘U-1’ compared to the corresponding level steps was found regardless of the gait posture with a significant increase of the ~20° in TF2 gait relative to the step ‘L’ (Figs 3F and 4B, Table 1). In the step ‘U0’, the Knee_*TO*_ decreased across gait postures. Trunk-flexed gaits demonstrated a significantly decreased knee flexion compared to the both steps ‘L’ and ‘U-1’ with no between posture differences (Figs 3F and 4C, Table 1). During the step ‘U+1’, participants increased their Knee_*TO*_ which was found to be significantly lower from those during ‘U-1’ and significantly higher from that of step ‘U0’ in trunk-flexed gaits. In this step, TF2 gait was associated with an increase of ~13° in Knee_*TO*_ compared with RE gait (Figs 3F and 4D, Table 1).

In the step ‘L’, trunk-flexed gaits demonstrated an increased Ankle_*TD*_ (Figs 3G and 4A, Table 1). Significantly increased ankle flexion (dorsiflexion) was observed in TF2 gait compared with RE gait in the step ‘U-1’(Figs 3G and 4B, Table 1). TF2 gait was associated with a significant increase of plantarflexion relative to the steps ‘L’ and ‘U-1’ but not significantly different from RE and TF1 gaits during the step ‘U0’(Figs 3G and 4C, Table 1). In the step ‘U+1’, Ankle_*TD*_ showed a significant increase only with respect to the step ‘U-1’ with no between posture differences (Figs 3G and 4D, Table 1).

As indicated by the analysis of simple main effects, during steps ‘L’ and ‘U-1’, no between step and between gait posture differences for CoM_*TD*_ were found (Figs 3H, 4A and 4B, Table 1). In the step ‘U0’, trunk-flexed gaits compared with step ‘U-1’ represented a significant increase of CoM_*TD*_ with a significant decrease of ~4% to 1.11 ± 0.03 from RE gait to TF2 gait (Figs 3H and 4C, Table 1). CoM_*TD*_ demonstrated a significant decrease in the step ‘U+1’ in all gait postures relative to the preceding corresponding steps with no between gait posture differences (Figs 3H and 4D, Table 1).

**Fig 5:**
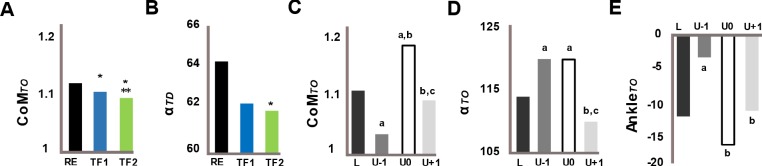
Main effects of posture and step. Shown are the main effects of posture on (A) CoM at TO and (B) leg angle at TD, and the main effect of step on (C) CoM, (D) leg angle and (E) ankle position at TO (N = 12). Significant differences from RE and TF1 are indicated with ‘*’ and ‘**’, respectively for the posture effect (p<0.05). Significant differences from ‘L’, ‘U-1’, and ‘U0’ are indicated with ‘a’, ‘b’, and ‘c’, respectively for the step effect (p<0.05). RE (black), regular erect trunk; TF1 (blue), *~*30° trunk flexion; TF2 (green), *~*50° trunk flexion; L, unperturbed level step (dark grey); U-1, pre-perturbation step (grey); U0, step-down (white); U+1, step-up (light grey).

Significant main effects of posture for the normalized vertical position of the CoM at TO (CoM_*TO*_) and the leg angle at TD (α_*TD*_) and of step for the CoM_*TO*_, the leg angle at TO (α_*TO*_) and the ankle joint at TO (Ankle_*TO*_) were found (Fig 5, Table 1). For posture factor, as compared to the RE gait, CoM_*TO*_ was decreased by ~2% in TF1 and by ~3% in TF2 (Fig 5A, Table 1), and leg angle at TD (α_*TD*_) was decreased by ~3° in TF2 (Fig 5B, Table 1). For the main effect of step, compared to the step ‘L’, while CoM_*TO*_ did not significantly change in the step ‘U+1’, in the step ‘U-1’ decreased by ~7% and increased by ~5% in the step ‘U0’ (Fig 5C, Table 1). α_*TO*_ was increased by 6° in the steps ‘U-1’ and ‘U0’. In the step ‘U+1’, α_*TO*_ was decreased by 10° relative to the steps ‘U-1’ and ‘U0’ and not significantly different from the step ‘L’ (Fig 5D, Table 1). Ankle_*TO*_ was decreased by ~9° in the step ‘U-1’ and was increased by ~14° and ~8° in the steps ‘U0’ and ‘U+1’, respectively, relative to the step ‘U-1’ (Fig 5E, Table 1).

## Discussion

Considering the frequent occurrence of trunk-flexed locomotion (e.g. in elderly and patients with spinal pathologies) and its detrimental effect on gait stability, understanding the role of the trunk in human locomotion is of clinical interest. In this study, we investigated the adaptive kinematic behavior of able-bodied walking while negotiating uneven ground with altered trunk orientations. In line with our hypotheses, we observed step-specific effects of posture on the kinematic behavior of able-bodied gait in most of the parameters of interest (Table 1). As compared with regular upright walking, trunk-flexed gaits across uneven ground exhibited: a) more crouched legs, characterized by sustained knee flexion during stance (Fig 4, Table 1), b) a greater TD-TO kinematic discrepancy in the effective leg (i.e. shorter legs at toe-off) (Fig 4, Table 1) and c) a marginally flatter leg angle at TD (Fig 5B, Table 1). Participants rotated their trunk backwards during step-down regardless of the trunk orientation (Fig 2C). A more pronounced trunk backwards rotation in trunk-flexed walking contributed to the observed elevated center of mass (CoM) trajectories during the step-down (Fig 4C) which may have facilitated drop negotiation. Finally, at the end of the step-up, participants restored the kinematic parameters to the level step values (Fig 4, Table 1), suggesting stability and robustness of the gait in able-bodied participants.

### Kinematic adaptations during the pre-perturbation step (U-1)

Our results partly supported our expectation of the step-specific effect of the trunk posture on the kinematic behavior of able-bodied walking in the pre-perturbation step. Compared to the unperturbed step, the participants demonstrated kinematic adjustments only in the effective leg length and knee angle at TO (Figs 3B, 3F and 4A, Table 1). In preparation to step-down, individuals increased their knee flexion, and the magnitude of the flexion was proportionally increased with an increase of the trunk flexion, which led to a shorter effective leg length at TO (Fig 4B, Table 1). In addition, the ankle angle tended to be more dorsiflexed (main effect) (Fig 5E). These kinematic adjustments in the lower limb resulted in a lower CoM position relative to the corresponding level steps (Figs 4B and 5C) in preparation to step down. This finding is consistent with a study by Muller et. al [9], who reported that at the end of the step before a visible drop during regular upright walking, individuals modulate their knee and ankle flexion which in turn leads to a lower vertical position of the CoM. Plus, the vertical position of the CoM lowered proportionally with an increase of the trunk flexion (Fig 5A).

Comparing the effective leg length between TO and TD in the pre-perturbation step to that of the unperturbed step, we observed a much shorter effective leg during the pre-perturbation step due to an increase of the trunk flexion (Fig 4B). In agreement with our previous study [5], where we reported a kinematic asymmetry in leg function, characterized by a longer effective length at TD than at TO when transforming posture from upright to almost horizontal orientation, here we found such discrepancy in the effective leg length with a pronounced difference in the preparatory step. The observed kinematic adjustments in the approach step seemed to be driven by the visual perception of the perturbation which may have allowed adaptive motor control strategies.

### Kinematic adaptations during step-down (U0)

Comprising approximately 50% of the total body mass [45], a deviation in the trunk orientation can have a significant effect on the position of the CoM and thus on human locomotion [5, 7, 14, 27]. Trunk kinematic adjustments during accommodating uneven ground can be influenced by the height of the drop and the availability of the visual guidance. In downward step on a camouflaged surface, the trunk backward rotation becomes larger than stepping into a visible drop and tends to increase proportionally with the drop height [9]. In both upright trunk gait with straight legs [9] and trunk-flexed gaits associated with crouched legs during traversing uneven ground, the trunk appears to reduce its angle in a compensatory fashion to diminish variations in the CoM position. The utilization of this mechanism with a more pronounced adaptation during trunk-flexed gaits resembles the small birds’ locomotion in exploiting their legs (i.e. a zig-zag-like configuration) to negotiate large terrain perturbations [47]. The backward rotation of the trunk as found in our young, healthy participants (Fig 2C) not only contributes to the significantly higher vertical position of the CoM relative to the pre-perturbation step across trunk-flexed gaits, but may counteract a potential increase in angular momentum during a step-down (Figs 3H and 4C, Table 1). This opens up new perspectives on the role of the trunk in locomotion, notably for specific populations e.g. elderly with a forward inclined trunk orientation [18, 48] or patients who display atypical trunk postures [49]. Thus, backward trunk rotation when dealing with step-down may reflect an adaptive strategy to enhance gait stability. To the best of our knowledge, no studies are available whether elderly or patients with an altered trunk posture already employ this strategy for negotiating downward steps in unassisted locomotion, e.g. when stepping down from a curb or walking down inclines.

In the present study, participants landed on a lowered level with almost no significant changes in the effective leg length (Figs 3A and 4C, Table 1). A more extended ankle compensated the more flexed knee; however, these kinematic adaptations in the step-down were not significantly different from their counterparts in unperturbed steps. In addition, an increase of the trunk flexion did not lead to significant changes in knee and ankle joints across gait postures (Figs 3E, 3G and 4C, Table 1). The only change occurred at the hip: the more flexed the trunk, the more flexed the hip at TD (Figs 3C and 4C, Table 1). While no step-dependent effects of posture on the leg angle at TD were observed, walking with 50° of trunk flexion (TF2) was associated with a flatter leg angle across steps (Fig 5B), possibly to compensate for the loss in the horizontal distance between the CoM and the CoP induced by a trunk flexion. Moreover, the standard deviation of plantarflexion was much higher for the step-down (U0) compared with the other steps, indicating that some participants used toe-landing at TD of step-down (Table 1).

The results partially confirmed our expectation for step-specific effects of posture at TO in the step-down. The leg configuration at TO was characterized by significant knee and ankle extension in order to elongate the leg and to facilitate the restoration of the CoM height during the following step-up (U+1) across gait postures (Figs 3B and 4C). The main effect of step at TO revealed a significant increase of leg angle (Fig 5D) and ankle plantar flexion (Fig 5E). The potential loss in CoM height due to an increased leg angle was overcompensated by the simultaneous elongation of the effective leg. In comparison to the preceding step and unperturbed level step, the discrepancy in effective leg length between TD and TO in the step U0 was minimized, as participants were attempting to launch themselves onto the elevated ground (Fig 4C).

### Kinematic adaptations during step-up (U+1)

In agreement with our expectation that step-specific effects of posture would occur and likely differ between TD and TO instants in the step U+1, individuals exhibited significantly different kinematic adaptations at TD from those of other steps (Figs 3 and 4D, Table 1). They landed on the elevated step (post-perturbation step) with a shortened effective leg at TD as compared to the corresponding unperturbed steps across gait postures (Figs 3A and 4D, Table 1). This observation was reflected in significant increases in the knee flexion across gait postures and a significant increased hip flexion during RE gait (Figs 3C and 4D, Table 1). A shortened effective leg length led to a lowered vertical position of the CoM across gait postures relative to the corresponding unperturbed steps; however, the vertical position of the CoM did not exhibit a significant change with an increase of the trunk flexion (Figs 3H and 4D, Table 1). The former finding can be attributed to a considerable flexion across lower limb joints (Table 1) and trunk (Fig 2C) (i.e. crouched posture) during RE gait, walking with a regular erect trunk, leading to a significant decrease in the CoM height while stepping up immediately after a visible step-down in ground. Therefore, the second expectation that kinematic adaptations would become more pronounced with an increase of the trunk flexion was weakly supported, as individuals attempted to accommodate the immediate recovery step from the perturbation during trunk-flexed gaits with a kinematic behavior that was not remarkably different from the upright walking. These findings suggest that kinematic adjustments in the global leg and CoM displacement in the step U+1 tended to be rather step-dependent than posture-dependent.

Remarkably, for each gait posture, the kinematic parameters returned to the mean values of the unperturbed corresponding steps at TO (Figs 3 and 4D, Table 1). This may have been facilitated by moderation of the CoM trajectory during step down (relative height of CoM increased significantly during step-down, diminishing absolute changes of CoM height), a strategy that has been suggested to be effective in improving the dynamic stability [50, 51]. The step-specific effects of posture on walking kinematic parameters indicate that modulation of the leg posture was necessary to achieve this. Considering that there were no significant changes in kinematic parameters comparing step-up and the level steps at TO for each gait posture (Table 1), we assume that the recovery of the gait was achieved at the end of the step-up, suggesting stability and robustness of the gait. This may have been facilitated by the sequence of step-down directly followed by step-up and the presence of the visual perception of the perturbation. We however do not know whether a comparable immediate recovery would be achieved when stepping down on a permanently lowered level. Moreover, having observed the kinematic strategy of backward trunk rotation during stepping down while adopting various trunk orientations alongside other step-specific global kinematic adjustments in able-bodied gait motivates examining the role of trunk movements in balance-compromised cohorts to see to what extent their control of trunk–accounting for nearly 50% of total body mass–might be different from that of able walkers.

## Conclusion

In summary, the results of the present study indicate that negotiating changes in ground level requires step-specific compensatory kinematic adaptations in lower limbs to maintain dynamic stability regardless of the trunk orientation. These adaptations occur not only at the end of the step-down, but also at TO of the pre-perturbation step and at TD of the step-up. Backward rotation of the trunk during step-down was not only a preventive strategy employed by able-bodied participants possibly to control forward horizontal and angular momentum of the body, but also to moderate changes in the CoM trajectory in trunk-flexed gaits. The young healthy participants recovered to steady gait in the step immediately following a downward step in ground even in the presence of trunk flexion. Trunk-flexed gait is associated with impaired postural control [18]. The incorporation of exercises with a greater focus on voluntary backward rotation of the trunk for negotiating step-down into fall-prevention intervention programs may be useful to enhance gait stability in patients and elderly who exhibit trunk-flexed postures during walking. Further perturbation experiments on humans with and without normal trunk posture in comparable conditions will be required to shed further light on the interaction between the trunk posture and locomotion.

## Abbreviations

CoM: centre of mass
CoP: centre of pressure
GRF: ground reaction force
L: unperturbed step in level ground
RE: regular erect trunk
TD: touchdown
TF1: ~30° of trunk flexion
TF2: ~50° of trunk flexion
TO: toe-off
U-1: pre-perturbation step in uneven ground
U0: step-down in uneven ground
U+1: step-up in uneven ground
